# Where’s the Disconnect? Exploring Pathways to Healthcare Coordinated for Youth Experiencing Homelessness in Toronto, Canada, Using Grounded Theory Methodology

**DOI:** 10.1177/10497323231208417

**Published:** 2023-11-10

**Authors:** Alzahra Hudani, Ronald Labonté, Sanni Yaya

**Affiliations:** 1 Interdisciplinary School of Health Sciences, 6363 University of Ottawa, Ottawa, ON, Canada; 2 School of Epidemiology and Public Health, 6363 University of Ottawa, Ottawa, ON, Canada; 3 School of International Development and Global Studies, 6363 University of Ottawa, Ottawa, ON, Canada; 4 The George Institute for Global Health, Imperial College London, London, UK

**Keywords:** emergency youth shelters, health system, hospital, engagement, healthcare coordination, youth homelessness, norms, resources, regulations, operations, systems thinking

## Abstract

About 900 youth experiencing homelessness (YEH) reside at an emergency youth shelter (EYS) in Toronto on any given night. Several EYSs offer access to healthcare based on youths’ needs, including access to primary care, and mental health and addictions support. However, youth also require healthcare from the broader health system, which is often challenging to navigate and access. Currently, little is known about healthcare coordination efforts between the EYS and health systems for YEH. Using grounded theory methodology, we interviewed 24 stakeholders and concurrently analyzed and compared data to explore pathways to healthcare coordinated for youth who reside at an EYS in Toronto. We also investigated fundamental parts (i.e., norms, resources, regulations, and operations) within the EYS and health systems that influence these pathways to healthcare using thematic analysis. A significant healthcare coordination gap was found between these two systems, typically when youth experience crises, often resulting in a recurring loop of transition and discharge between EYSs and hospitals. Several parts within each system act interdependently in hindering adequate healthcare coordination between the EYS and health systems. Incorporating training for system staff on how to effectively coordinate healthcare and work with homeless populations who have complex health needs, and rethinking information-sharing policies within circles of care are examples of how system parts can be targeted to improve healthcare coordination for YEH. Establishing multidisciplinary healthcare teams specialized to serve the complex needs of YEH may also improve healthcare coordination between systems, and access and quality of healthcare for this population.

## Background

Approximately 11% of the homeless population in Toronto, Canada, is comprised of youth aged 16–24 (City of Toronto, 2021). Youth experiencing homelessness (YEH) are exposed to several factors associated with poor health including inadequate nutrition, increased risk of injury, and increased exposure to a range of infectious diseases (Boivin et al., 2005; Gaetz et al., 2010; Roy et al., 2004). Among these factors, poor mental health and addictions are the most prevalent (Kidd et al., 2017). Many YEH suffer from addictions and chronic mental health issues including severe mental distress, depression, anxiety, and suicidal ideation (Kidd et al., 2018; Kulik et al., 2011). Poor mental health is often associated with childhood trauma, discrimination, and the experience of homelessness itself (Kidd et al., 2018). Findings from the second and most recent national youth homelessness survey (2019) (*N* = 1375) indicate that 74% of youth respondents report being highly distressed, 35% report attempting suicide at least once, and 33% report a drug overdose requiring hospitalization (Kidd et al., 2021). Of those youth suffering from mental illness, 60% present with multiple diagnoses (Slesnick & Prestopnik, 2005). Several studies indicate a dose–response relationship between exposure to homelessness and mental health decline (Hadland et al., 2011; Kidd et al., 2017). Findings from the 2015 and 2019 national surveys also indicate that about 25–30% of youth seeking shelter reside at an emergency youth shelter (EYS) in Canada (Gaetz et al., 2016; Kidd et al., 2017, 2021).

Approximately 900 youth between the ages of 16–24 reside at an EYS in Toronto on any given night (City of Toronto, 2021; Noble et al., 2022). Although EYSs are mandated to provide support for youth’s immediate needs, they have evolved to offer programs and services to help improve youth’s health and determinants of health (e.g., therapy, housing support, and employment assistance programs) (Karabanow, 2002). While these services are convenient for youth to access on-site, they are not uniformly offered across EYSs in Toronto, largely due to limited funding and resources within the sector. Further, youth suffering from complex and chronic health issues may require care from the broader health system, where they can readily access advanced health technology for their emergency and/or specialized health needs (Gaetz, 2014). For youth to receive optimal healthcare, shelter and health system staff must work collaboratively to coordinate healthcare effectively (Hwang & Burns, 2014).

Currently, it is unknown how EYS and health system staff who work within their siloed systems support youth with coordinating care for their health needs. Understanding youth’s pathways to healthcare as coordinated by system staff, and barriers preventing staff from seamlessly coordinating healthcare for and/or with youth, may enable stakeholders within Toronto’s EYS and health systems with a starting point from which to collectively target various system elements to improve healthcare coordination for YEH.

The research questions guiding this study were (1) *How is healthcare coordinated for youth residing at EYSs in Toronto?* and (2) *What factors influence youth’s trajectories to healthcare?* In responding to these research questions, we aimed to (1) outline distinct pathways to healthcare for youth who reside at EYSs in Toronto and (2) analyze and discuss various system parts and characteristics that influence these pathways to healthcare within and between the EYS system and health system. This study was informed by a systems thinking and organizational change framework, where we breakdown fundamental parts of each system that may be influencing the current state of healthcare coordination for youth residing at EYSs in Toronto.

## Methods

### Study Design

This research is part of a larger case study exploring how the EYS system and health system engage to coordinate healthcare for YEH within the inner-city and inner suburban regions of Toronto. The methods used to respond to the research questions in this study are twofold and consist of those that align with constructivist grounded theory methodology and thematic analysis. We first employ constructivist grounded theory methodology to understand intra- and inter-systemic healthcare coordination processes that influence youth’s trajectories to healthcare within and between the EYS and health systems, and in doing so inductively generate a theoretical explanation about this complex and lesser-known phenomenon. Charmaz’s constructivist approach assumes a relativist epistemology and acknowledges the roles of the researcher and interview participants in the construction and interpretation of the data (Charmaz, 2017).

We then analyzed these pathways further using thematic analysis to understand the fundamental system parts (i.e., system-based norms, resources, regulations, and operations) that affect healthcare coordination processes and quality within and between the EYS and health systems and how these parts might influence youth’s pathways to healthcare. Thematic analysis of the fundamental system parts (see Table 1) and their roles in healthcare coordination for YEH are approached using essentialist/realist epistemology (Clarke & Braun, 2017) and are guided by the second component of Foster-Fishman and colleagues’ theoretical framework for transformative systems change (Foster-Fishman et al., 2007). These fundamental system parts are critical to explore as they affect the relationships that YEH have with organizations and staff within each system.

**Table 1.** Fundamental System Parts.

System parts	Description
Norms	System norms refer to the attitudes, beliefs, and values held by organizations within systems, which can dictate behaviours. When these worldviews are shared by systems or subsystems, they play a role in determining their policies, practices, and functions.
Resources	System resources encompass human, social, economic, and opportunity capital.
	• Human resources include the knowledge, skills, and abilities that exist within the system.
	• Social resources consist of the relationships between system staff which would thereby influence system functioning—i.e., how healthcare is coordinated within and between systems.
	• Economic and opportunity capital refer to the distribution of financial and organizational resources to facilitate improved coordination of healthcare.
Regulations	System regulations refer to the policies, procedures, and routines that exist in coordinating healthcare for youth.
Operations	System operations refer to power and decision-making within systems—understanding this can help identify root causes of gaps within systems, as they relate to coordinating healthcare for YEH.

### Data Collection

This qualitative study comprises interviews with key informants involved at various levels of the EYS and health systems in Toronto and YEH who have navigated healthcare while residing at an EYS. Twenty-four in-depth, semi-structured interviews were facilitated virtually with key informants between May 2021 and March 2022. First, we used purposive sampling to recruit high-level executives and clinicians who worked within the EYS system and health system, respectively. We then used snowball sampling to recruit other stakeholders including lower-level executives (e.g., case managers) and various frontline staff with clinical (e.g., registered nurse and social worker) and non-clinical (e.g., program coordinators and outreach counsellors) backgrounds. A few frontline staff working at EYSs and within the community health sector supported youth recruitment by posting flyers at their respective organizations or personally sharing flyers and information sheets with youth who met inclusion criteria. Youth were interviewed if they ranged between 16 and 24 years of age, resided at an EYS in Toronto, and navigated healthcare integrated within an EYS or the broader health system. In total, we interviewed eight key informants employed within the EYS system, seven within the health system, three whose work overlapped across systems, and six youth.

Two separate interview guides were developed for youth and non-youth participants. Interview questions were open-ended and focused on identifying and assessing healthcare coordination processes within and between the EYS and health systems in Toronto and fundamental system parts that influence these processes.

### Data Analysis

#### Grounded Theory

Interview transcripts were filed into NVivo 12.0 software and inductively coded to identify and construct pathways to healthcare taken by YEH. Three layers of coding were applied based on Charmaz’s constructivist grounded theory approach (Charmaz, 2017). First, we used open coding to identify actions and processes relevant to coordinating healthcare within and between systems. Open coding took place sentence by sentence, and in some cases segment by segment, and included gerunds to label actions and processes relayed by key informants. We then used focused coding to categorize predominant initial codes into defined categories. Finally, theoretical coding was used to integrate these categories and illustrate youth’s trajectories to healthcare. Analytic memos were documented simultaneously post-interviews and while analyzing data. Memos included descriptive summaries of interviews; emerging patterns, categories, and/or concepts; and personal thoughts and reflections related to the study. Interview data were constantly compared to identify healthcare coordination processes mentioned by interview participants. The interrelationship between the coding process, analytic memo writing, and constant comparative analysis helped formulate the grounded theory.

#### Thematic Analysis

Thematic analysis was then used to identify, analyze, and interpret critical system parts associated with healthcare coordination processes within and between systems (Clarke & Braun, 2017). We used deductive, theory-driven coding to organize interview data into predetermined categories based on the fundamental system parts (systems norms, resources, regulations, and operations) and sub-categories that fall under these parts, as described in Table 1. Inductive coding was used to further identify semantic themes within these categories and sub-categories to explicate how they affect youth’s pathways to healthcare within and between the EYS and health systems. Thematic analysis was also conducted using NVivo 12.0 software.

### Ethical Approval and Considerations

Ethical approval was granted by the University of Ottawa Health Sciences and Sciences Research Ethics Board. Non-youth key informants were provided with an informed consent form to review in advance of their interview. Youth were e-mailed a 1-page information sheet and flyer outlining important study details, after which they could contact the principal investigator (AH) with any questions and/or schedule the interview. If youth preferred, AH offered this information over the phone. Written or verbal consent was provided by all key informants who agreed to participate in an interview. Verbal consent and interviews were recorded and transcribed using Otter.ti software. Youth were compensated $30 through e-transfer for their time. Further, AH is trained in structuring safety when interviewing populations who have experienced trauma (Reynolds, 2014). She ensured that all youth participants had a safe and private space to participate in the interview and assured them of their anonymity in any knowledge dissemination products materializing from this work.

### Trustworthiness

Rigor in this study was achieved by embracing the core practices of grounded theory methodology. This includes constant comparison of emerging data; detailed and reflexive memo writing to articulate healthcare coordination processes and personal reflection; three layers of coding; collecting data until reaching theoretical saturation; and member checking pathways to healthcare with six key informants who work within either system. Confirmability was enhanced through data triangulation (Nowell et al., 2017). Additionally, themes discovered through thematic analysis aligned with several memos that were previously documented.

## Results

**Figure 1.**
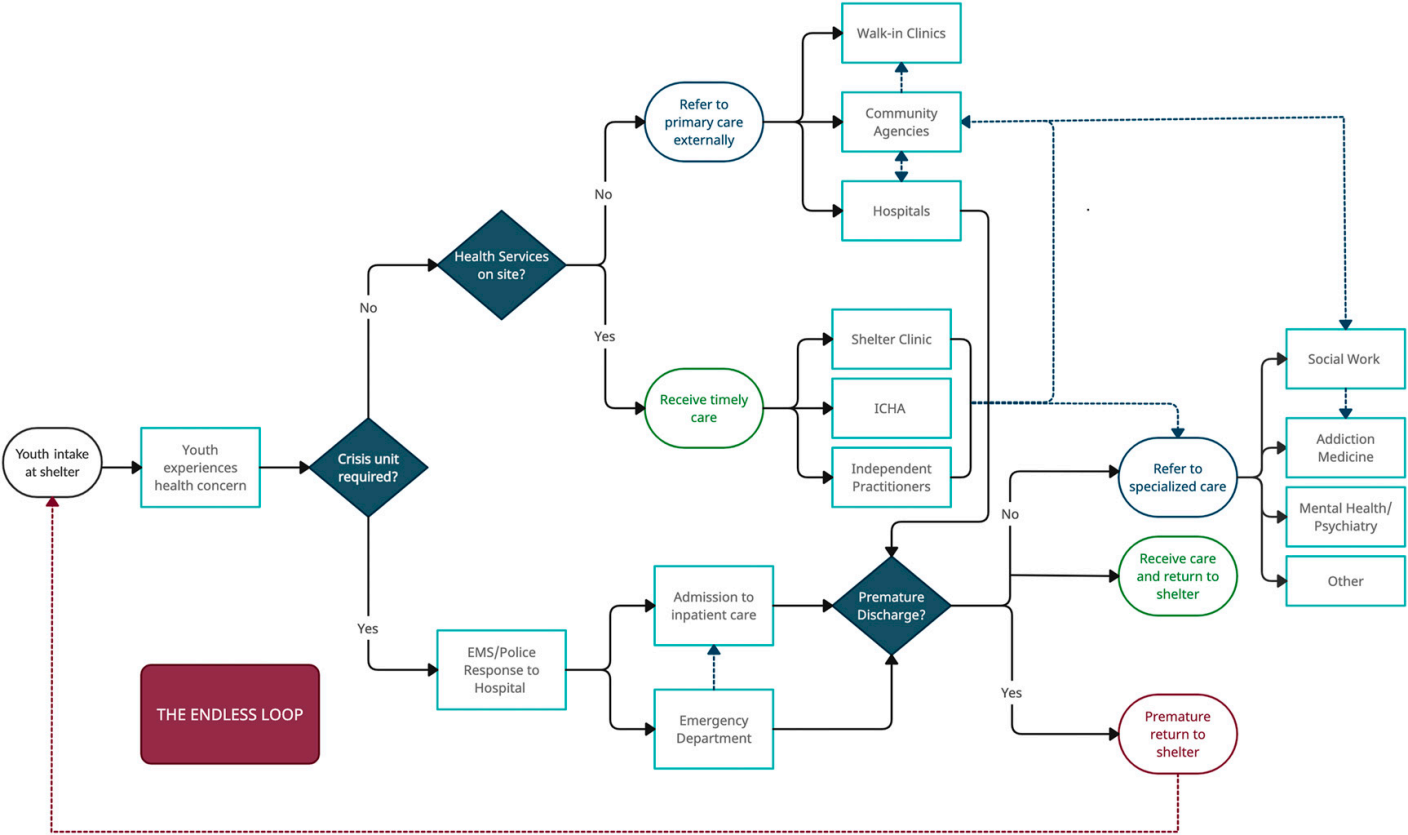
Pathways to healthcare process map.

Youth residing at EYSs in Toronto follow various pathways to healthcare depending on their needs and the healthcare resources integrated at EYSs or within the health system (see Figure 1). Since policies or protocols on healthcare coordination between the EYS and health systems do not exist, staff often use their own professional training, internal health and safety protocols, and relationships developed with external organizations to provide or coordinate health services for their youth clients or patients.

Most youth access care within the health system independently when experiencing health concerns. However, support with coordinating healthcare by or with system staff can help youth access care within the complex health system, which can be challenging to navigate alone. EYS staff support with coordinating healthcare for their youth clients if they are aware of their health needs and receive consent or if youth are experiencing crises they are not equipped to care for (e.g., overdose and suicide attempt). If youth are not prematurely discharged from hospitals and successfully progress to receive specialized care (if needed), healthcare coordination is typically led by hospital staff. The maroon dashed arrow represents a critical disconnect in the pathways to healthcare—the endless shelter–hospital loop often implicating the most vulnerable youth. The navy-blue dashed arrows represent possible routes to healthcare, as referred by healthcare providers within the health system. The grounded theory proposed is depicted through Figure 1 and highlights how insufficient engagement and poor healthcare coordination within and between the EYS and health systems can lead to youth’s entrapment in the endless shelter–hospital loop—a possible outcome through multiple routes in youth’s trajectories to healthcare. Higher risk youth who reside at EYSs with fewer healthcare resources and healthcare coordination capacity often end up taking the shortest route to possible premature discharge leading to the endless loop. Each system is dissected further to provide context on the norms, resources, regulations, and operations that affect healthcare coordination and inevitably youth’s distinct trajectories to healthcare.

## Factors Affecting Pathways to Healthcare

### System Norms

Organizational missions and values and the personal attitudes, beliefs, and values of staff influence youth’s pathways to healthcare. Staff within EYSs and community-based agencies (e.g., health centres, drop-in programs for YEH, and Inner City Health Associate (ICHA) clinics) commonly describe the importance of engaging in trauma-informed, youth-led, and youth-focused care and practicing resiliency and strengths-based approaches to care. An ICHA physician describes why:

*“I think having trauma-informed care is really important because a majority of our youth have experienced an incredible amount of trauma and have sometimes had negative experiences with the healthcare system. I also think providing non-judgmental and low barrier care and making it very clear that it’s a safe environment [are important] ... letting the youth know that anything they don’t want to talk about or engage in is completely fine.”*

When youth feel safe and feel that they can trust the system and staff, they are more likely to communicate their needs to their caseworkers and follow non-crises routes to healthcare.

Within hospitals, core values include evidence-based care, recovery-oriented care, and serving with compassion. However, an emergency department physician explains, *“Although compassion is one of the values advertised, I think it wavers and obviously depends on the experience level of the provider, and there could be a certain point where people have compassion fatigue.”* Several YEH describe feeling unwelcome, unheard, unsupported, and uncared for by healthcare providers at hospitals. A 19-year-old youth explains how hospital care can be improved based on her experience: *“If they actually took the time to listen, not go into their weird doctor mode, and talk to me like I’m an actual fucking person.”* Shelter staff, community healthcare staff, and youth informants agree that YEH do not always receive the care and attention they need at hospitals, often leading to the recurring shelter–hospital loop.

Staff who work at hospitals and have a background in working with homeless populations demonstrate similar values to EYS and community health staff. For instance, a staff member involved in a hospital program dedicated to supporting people experiencing homelessness discusses the importance of dignified and equitable care for all, being person-centered, and aligning with trauma-informed and harm reduction philosophies. Other staff involved in similar initiatives discuss their ability to identify the needs of YEH and pair it with a resource—this includes coordinating care for YEH beyond their visit to the hospital. For example, a coordinator working within the emergency department mentions connecting patients to health services through Coordinated Access to Care for the Homeless (CATCH) referrals—a short-term case management service developed in collaboration with ICHA, St. Michael’s hospital, and Toronto North Support Services to link individuals experiencing homelessness with psychiatry, primary care, and initiating housing plans. Her ability to help patients navigate healthcare depends significantly on her attitude towards them. She explains, *“At the core of it is relationships and trying to see somebody through the entirety of who they are and not just the situation they’re in … then you’re allowed to navigate.”* Building trust with people experiencing homelessness is a critical first step to supporting them with their healthcare coordination needs, whether through the EYS or health systems.

### System Resources

#### Human Capital: What Knowledge, Skills, and Training Exist and Are Needed Within Each System?

Most EYS staff believe they have the skills and knowledge needed to coordinate healthcare well—this includes applying a trauma-informed approach to care and supporting YEH navigate community resources. Insufficient funding and human capital were stated as factors hindering healthcare coordination within and between systems, even if staff held attitudes and values that supported work with YEH. An executive at Shelter C shares, *“Shelters may benefit from having more staff, 40:2 is a pretty big ratio for youth to staff … we cannot meet the [Toronto Shelter] standards without adequate funding.”*

EYSs sampled in this study employ a general case management approach to coordinating healthcare for youth. Most staff discuss being trained on the job, shadowing others, and learning from response to crisis and health concerns commonly experienced by youth. Additionally, the educational and occupational backgrounds of shelter and hospital staff significantly contribute to how healthcare is coordinated for youth. An executive at Shelter A shares:

*“Registered nurses have training that they [get] through nursing school, the doctor, same thing … case managers, some of them have a social work background, so would have a case management approach grounded in social work practice, but there isn’t like one formal process for coordinated care.”*

Staff who typically coordinate healthcare and have observed gaps and challenges with them agree that staff within both systems could benefit from receiving joint training. A clinical manager at Shelter A states, *“There should be training for us, and teams at the emergency departments, together—so that things are coordinated. Sure, we can have training [for] coordination of care, but what does it mean if other teams aren’t working consistently.”* Frontline staff at hospitals commented on how training focused on serving gender-diverse youth, harm reduction, trauma-informed care, and anti-oppression could help effectively serve homeless youth populations who frequently visit inner-city hospitals. Currently, there is no training or education that helps hospital healthcare providers situate or understand the contexts of this population. Further, EYS and hospital staff agree that being aware of relevant health services in their region is necessary to coordinate healthcare well.

Hospital-based healthcare providers mention having few or no relationships with EYS staff. A social worker at a Toronto hospital discusses taking the initiative to create a list of resources through the 519 to share with her youth patients to access independently. Moreover, most hospital referrals are coordinated internally. A few healthcare staff discuss the benefit of knowing how to connect youth to community health and housing resources and contacting the central access line, but also limitations that may exist with external community-based referrals. A counsellor at a Toronto hospital shares, *“There’s nothing we can do except make community referrals and cross your fingers and toes that those connections and referrals actually work out because once people are discharged, you can’t do anything.”* This is a significant reason why relationship-building and navigation support through hospitals and with EYS providers can help improve healthcare coordination between systems.

#### Social Resources: What Relationships and Interactions Exist Within and Between Systems?

Strong inter-organizational relationships allow for important information and resources to diffuse through each system and support the development and transfer of norms, attitudes, and knowledge for effectively coordinating healthcare for YEH (Bailey & Koney, 2000). While intra-organizational relationships within the EYS and health system are reportedly strong, inter-organizational relationships within and between these systems are generally weak and depend significantly on pre-existing connections between high-level executives and healthcare providers. A clinician at Shelter A shares:

*“I think the shelter sector generally needs to work together more. I think part of it goes to the fact that people are vying for the same pools of money and so sometimes that creates competition rather than a sharing of resources … other than ICHA physicians also going to [Shelter B] and coming to us, and those are actually different physicians, we don’t share healthcare resources.”*

The Youth Shelter Interagency Network was noted as a key platform through which shelter leadership convened regularly to discuss any processes, learnings, and challenges related to coordinating healthcare for YEH.

Most relationships developed between EYSs and the health system are described as informal, unless integrated on-site or formally established. Shelter staff frequently refer youth to external healthcare services or connect with individuals in their networks who can help improve processes of coordinating healthcare between systems. EYS staff agree that they are more successful in establishing relationships with healthcare organizations that have similar aims and values as them, typically community agencies. Establishing strong, positive relationships with hospitals have generally been challenging for EYS staff. Similarly, hospital staff did not report any strong working relationships with EYSs in their region unless initiated by staff involved in specific programs focused on improving the health outcomes of homeless populations.

#### Economic and Opportunity Capital

The underlying challenge with economic and opportunity capital boils down to critical underfunding within the EYS system and a scarcity of shelter, supportive housing, and targeted health services for YEH. When asked about how the two systems can use their respective resources differently to improve healthcare coordination, the consensus was that there is a need for more resources overall. Most interview participants believed that little to no money was wasted within either system, but rather the larger issue was having to fund crisis response as opposed to prevention. The term “zero-sum game” was used analogously by a hospital researcher and clinician to describe the possible outcome of redistributing financial resources within systems to improve healthcare coordination. He elaborates:

*“You can’t stop funding crisis response because people will suffer and die, but at the same time, you need to move resources to prevention. So, for a period, you need to do both, which is expensive—you need to fund prevention, so things like housing first, family reconnect, etc... an upstream response, and that will start to reduce the flow of youth into crises. As you do that, you will need less crises services, fewer shelter beds, you can start to take money out of that ... and fewer emergency hospitalizations. Then the money, the demand there starts to go down…“*

Other propositions to reduce recurring emergency department visits and thereby financial strain on the health system include (1) integrating health services within each EYS; (2) developing meaningful connections between youth and health services within the health system; and (3) establishing targeted health services in the community, aiding youth with community reintegration (e.g., Youth Wellness Hubs).

### System Regulations

Currently, there are no written policies, procedures, or training offered across systems on coordinating healthcare for YEH. A local government executive confirms, *“Training specifically on the integration [HC] piece is a good question. I can’t think of any specific training that we have in place for that.”* For many EYS staff, healthcare coordination is *“learnt on the job.”* Despite this, in-house processes for coordinating healthcare are similar across EYSs and some health system organizations as depicted in Figure 1, although executed by different staff within each system, depending on their capacities. For example, a shelter with an in-house, nurse-led clinic may have nurses lead efforts to coordinate healthcare for youth, whereas shelters who do not have such infrastructure may delegate this task to other staff on their team, such as a psychologist, case manager, or youth worker.

Efforts to coordinate healthcare for youth residing at EYSs are usually successful once trusting relationships are established between youth and staff, and once youth provide consent. Several lines of communication exist internally, where staff communicate youth’s health information to team members, for example, resources they have been referred to and medication they require. Barriers in coordinating healthcare increase when youth are sent elsewhere to receive care—especially to hospitals when experiencing crises. The largest barrier in coordinating healthcare for youth in these circumstances is poor communication between system staff, largely due to privacy concerns and information-sharing policies preventing staff from discussing critical information required to support continuity of healthcare following hospital discharge. Additionally, youth often return to EYSs without discharge papers making it challenging for EYS staff to support and/or coordinate next steps in healthcare. YEH also face barriers in communicating crucial information to EYS staff and hospital healthcare providers when admitted to hospitals.

#### Information-Sharing and Privacy Policies

Information-sharing and privacy policies within each sector prevent staff from exchanging important information and following up about youth’s health status and/or next steps in healthcare, although technically considered circle of care. This gap in follow-up sometimes results in (1) youth losing their bed and belongings when returning to shelter from a prolonged hospital stay, usually in a fragile state, and (2) EYS staff being unable to convey essential information to hospital healthcare providers during crises. Below are two scenarios describing these common, spiralling challenges.

Scenario 1: A manager at Shelter A shares:

*“If youth are residing in shelter, we’ll hold their bed for up to five days—this is mandated by the city. So, because it’s mandated by the city, we can’t hold their bed longer than that, we [will] have to give it up because shelters are frequently full. So, when I send a young person [to the emergency department], I will then call that emerge or patient locating, and speak to the provider, whether it’s a physician or registered nurse who’s caring for our client, and just say, “Hey, I’m from [Shelter A], and I want to know ... these are our bed hold policies so let me know if you’re planning on keeping them longer, if so then we’ll have to pull the bed. If you think it’s going to be a few more, day maybe days, we can appeal the city.” And I want to be very respectful of their privacy policies. But we’re seeing a huge discrepancy in how different hospitals and different staff operate.“*

Scenario 2: A community health clinic manager shares an example of how these policies can lead to barriers in supporting healthcare post–hospital discharge:

*“Unfortunately, the patient was discharged [from the hospital mental health department] with medication that wasn’t covered—there was a lot back and forth to see how we can get this medication covered because this patient didn’t have any identification, let alone coverage. Unfortunately, we couldn’t get them access to the medication. And it was disappointing to see that happen.”*

Instances like these often contribute to the recurring shelter–hospital loop, even when youth are referred to hospitals within the health system through community health agencies. Overall, the lack of communication between shelter and hospital staff significantly contributes to youth’s entrapment in the endless loop. Moreover, EYS and hospital discharge policies also contribute to the endless loop, impeding youth’s escape from the vicious cycle of homelessness and poor health.

#### Discharge Policies and Procedures

##### How Do Shelter Discharge Policies Get in the Way?

EYS discharge policies can interfere with youth receiving critical healthcare and/or supporting with coordinating healthcare for or with YEH. Discharge policies include rigid rules around chores, curfew, and being caught with substances on-site. A 24-year-old newcomer man suffering from a neurological disorder was late to his surgery because he was asked to complete his chores before leaving the shelter. He shares:

*“On the day of my surgery, I was late to the hospital. But that’s because the worker at the shelter wanted me to do a chore. I tried to tell her that I have surgery, but there are rules right, if you don’t do a chore, they will give you a warning. And according to the rules ... if you get three warnings, you get discharged.”*

Post-surgery, this young man lost his shelter bed and belongings due to the shelter bed-hold policy:

*“I lost my bed, suitcase and everything because I was in the hospital for a week. And I wasn’t able to call, so yeah, as a newcomer it was tough to lose everything that belonged to me.”*

Other youth shared similar stories about losing their bed in times when they may have needed one most.

A clinician working at Shelter B agrees that shelter policies need to be rethought. When asked about policies that hinder healthcare coordination, she shares:

*“That’s been my biggest challenge, if a youth gets three warnings they are discharged, and the warnings could be because they were rude, or they refused to do a chore. A lot of times it’s youth that are just new to the shelter. And so, they’re coming in, who knows the circumstances under which they came from, and they’re struggling, and they break. A lot of times they’re angry at the world, they’re angry at everybody. And I just find that this is the youth that needs a lot of support. Not, you know, “don’t talk to me like that, that’s your third warning. Go pack your bags, you’re out of here” … discharges should only be the health and safety of the youth, other youth, or staff.”*

Enforcing such policies conflicts with the core value of trauma-informed care at EYSs.

##### Premature Discharge From Hospitals: What Happens and Why?

YEH are often prematurely discharged from hospitals. This occurs when they (1) experience stigma and discrimination by hospital staff; (2) are escorted out by security prior to receiving medical care; and/or (3) have mental health concerns that are commonly dismissed by healthcare providers. A 19-year-old woman suffering from depression, anxiety, and addictions shares her experience:

*“It’s hard to just walk into a hospital and say that your brain is all fucked up. Or to walk into a walk-in clinic and say that ... sometimes the way they treat you changes, if they find out that you are a sheltered kid, especially if you are going in for substance abuse. At that point, it doesn’t matter that you needed help, or felt like you were going to die—just like you did this to yourself, you need to leave.”*

She also discusses facing mental health comorbidities that have been dismissed and exacerbated during her stay at the hospital:

*“I’ll tell them, just let me be here overnight, let me stay till morning. And then [it becomes] a whole thing and makes it worse and my mental health kicks in because I have all these different disorders and that doesn’t help. If you don’t freak out, they won’t believe you unless you actually physically tell them that you’re going to kill yourself. It’s like, I’m not suicidal. Like, can I hurt myself? Or others? Yes. Doesn’t mean I want to, and then they just wait for that to happen, before saying, okay, come back.”*

An executive at Shelter C, who has over 20 years of experience working within the EYS system, shares her observations on why premature discharge from hospitals happen, particularly in the context of mental health issues:

*“One of the things that often happens is, we’ll have a young person who has thoughts of suicide, and off they go to the hospital, the hospital thinks it’s not valid and then back they come to us, and then they may have those thoughts again, and off they go back to the hospital and then back they come to us—and that dance has been at every single shelter that I’ve worked at. And so, I think we should, err on this side of caution, when sending a person to get assessed—but also when a person goes to the hospital, they don’t want to stay there, they may not be treated well, they may not be taken seriously.”*

### System Operations

#### Crises Versus Non-Crises Routes to Healthcare

Power in decision-making to coordinate healthcare depends significantly on whether youth are experiencing crises that require emergency care. The executive at Shelter C shares,

*“I think our role is to identify if we think this person is either in danger or a danger, get them to the health system and then the health system decides whether or not what we see is valid and requires an assessment.”*

In non-crises situations, stakeholders at various levels within both systems have the power to influence healthcare coordination for YEH. The hierarchy in decision-making starts with the provincial and local governments. The Ministry of Health has the power to develop relevant policies and enforce integrated programs and services for this population, and the City of Toronto controls the delivery of base shelter programming and support, including any frameworks or practices to improve healthcare coordination or health service integration within EYSs. A case manager at Shelter B shares, *“We are a city run shelter at the end of the day, and coordination of care between different shelters, and shelters and other providers is trenched within the city’s policies, as well as our private agency policy.”*

At subsequent levels, decision-making in healthcare coordination is influenced by EYS or health system leadership and/or staff providing or coordinating healthcare depending on youth’s position in their trajectory to healthcare. EYS executives and leaders are responsible for deciding how fundraised money is spent in efforts to coordinate healthcare for YEH: whether they are spending resources developing inter-organizational relationships with independent practitioners and/or health agencies to integrate health services on-site or referring youth to community-based healthcare. Additionally, efforts are made to advocate for youth when observing gaps in their care. Clinical staff at shelters and hospitals sometimes inadvertently act as liaisons between systems by offering to connect youth to other healthcare services based on their needs.

Most EYS and health system staff believe that youth lead decision-making in coordinating care for their health needs, as they must consent to receive care. A few EYS staff also discuss the importance of involving youth in decision-making for shelter programming and healthcare to develop effective models of integrated and coordinated healthcare. An executive at Shelter A states, *“I think working with young people needs to really emphasize co-design, engaging young people, and understanding what type of shelter they want, how they want services delivered, etc. Does that happen? Yes. Does that happen enough? Probably not.”*

## Discussion

In addition to being supported with healthcare coordination, youth may involuntarily have healthcare coordinated for them by staff (e.g., being formed at an EYS) and/or choose not to seek healthcare at various points of non-crises pathways, especially if they experienced the endless loop and have “given up” on accessing health services. One of the biggest challenges identified in youth’s trajectories to healthcare is premature discharge from hospitals during crises, often resulting in an endless loop between shelters and hospitals. Various elements within these fragmented systems feed this recurring pathway—a few of which include health system norms, which may result from insufficient staff knowledge and training for serving marginalized populations experiencing homelessness, who may have complex mental health needs; information-sharing and privacy policies preventing important communication exchange between system staff; and having only few formal inter-organizational relationships between systems through which to coordinate healthcare appropriately and effectively. Further, we found that coordinated pathways to mental healthcare and addictions support for youth are scarce and barely identified—another factor leading to the endless loop between systems. This is especially important given the high prevalence of mental health issues and addictions among YEH. According to organizational theorists, constructive change within systems will only occur if the deeper structures within systems (i.e., fundamental system parts) that contribute to poor systems functioning are targeted (Gersick, 1991). These system parts are significantly interdependent, as demonstrated in this study.

Integrating accessible and timely primary and mental healthcare on-site at EYSs was proposed by several KIs to improve access to healthcare for youth and encourage more non-crises routes to care. Specialized healthcare approaches in the United Kingdom and the United States demonstrate additional pathways to integrated healthcare for homeless populations (Davies & Wood, 2018). Findings from a randomized controlled trial in the United Kingdom demonstrates that specialized general practitioner–enhanced care at hospitals for the complex needs of homeless patients improved patients’ quality of life post-discharge (EQ-5D-5L score increased by .12 [95% CI .032 to .22] in the enhanced care arm compared to .03 [−.1 to .15; *p* = .076] with standard care) (Dorney-Smith et al., 2016; Hewett et al., 2016). Additionally, the Healthcare for Homeless model embedded in the US federal healthcare system emphasizes a multidisciplinary approach to coordinating healthcare in collaboration with community healthcare providers and social service agencies (Zlotnick et al., 2013).

Nonetheless, the recent establishment of Ontario Health Teams in Toronto, while broadly targeting all-encompassing population health needs (Bond, 2019), is similarly promising in helping youth at EYSs who have complex health needs navigate healthcare with the support of a professional, multidisciplinary team. To our knowledge, multidisciplinary healthcare teams specialized to serve the complex needs of YEH or other homeless populations within Toronto-based hospitals do not exist, although many youth go or are sent to hospitals to receive emergency care. Such an initiative would require teams consisting of diverse healthcare providers across primary, secondary, and community care. Teams would require adequate training to appropriately build trust and rapport with individuals experiencing homelessness; knowledge and awareness of resources within both systems; sufficient follow-up with clients; adequate communication across integrated and external care providers; and assistance with housing programs/housing first (Dorney-Smith et al., 2016). Hospital stays present an opportunity to engage YEH with secondary care, and community healthcare and services which can help improve other aspects of their health and determinants of health. We recommend that such integrated pathways to healthcare be considered for study by clinician-researchers in Toronto, who may be affiliated with major inner-city hospitals and health centres. Current programs supporting homeless populations with systems navigation is a step in the right direction. Additionally, efforts to involve YEH as peers within EYS and health system programming are recommended based on favourable evidence from recent programs (Kidd et al., 2019; Stewart et al., 2009). Additionally, like the US Healthcare for Homeless model, healthcare coordination within Canadian systems may be improved significantly from implementing and enforcing information-sharing through electronic medical records across the EYS and health systems (Angoff et al., 2019)—this will enable service providers within circles of care to adequately follow youth’s healthcare trajectories whether accessing integrated healthcare at shelters or that within the broader health system.

### Future Directions

The next steps in this case study are to integrate these system elements and assess their interactions in current healthcare coordination efforts. A causal loop diagram will help visualize balancing and reinforcing interdependencies and feedback within and between systems. The aim is to identify in detail which areas within the EYS and health systems can be targeted to improve healthcare coordination for YEH.

### Strengths and Limitations

Triangulating data from stakeholders at various levels of the EYS and health systems, including YEH, helped to develop a comprehensive overview of pathways to healthcare coordinated for YEH in Toronto. The theoretical framework for transformative system change informed the interview guides and helped uncover deep structures within these systems that would benefit from being explored further by researchers, policymakers, and system leaders. A major limitation to the study is the small number of key informants recruited from the large, complex health system. Despite our repeated attempts to recruit health system staff, few healthcare providers were responsive or available to interview—largely due to the health system burden caused by the COVID-19 pandemic. Interviews with walk-in-clinic staff and more hospital and community health centre staff would help enhance confidence in the process map developed based on interview data. Moreover, involvement in Ontario Health Teams was mentioned by some informants during interviews—however, it was too early to capture the impacts of these healthcare coordination and systems integration efforts.

## Conclusion

Youth’s pathways to healthcare as coordinated by EYS and health system staff depend significantly on the independent and interdependent role of norms, resources, regulations, and operations built into each system. Decision-makers across the EYS and health systems, including YEH, must work collaboratively to develop protocols, policies, and training that will help establish and improve pathways to coordinated healthcare for youth. Amending policies to improve communication and discharge processes within and between systems may reduce the frequency of youth who commonly get trapped in the EYS–hospital loop. Overall, we recommend that decision-makers engage YEH in efforts to strengthen healthcare coordination within and between systems, particularly by targeting some or all the fundamental system parts discussed.

## Note

1. The sentence “All key informants who were interviewed provided verbal or written consent prior to their interview” was removed from Ethics Approval section and added at the start of the Consent to Participate section after the article’s original OnlineFirst publication.

## Appendix

### List of Abbreviations

CAMH: Centre for Addiction and Mental Health
CATCH: Coordinated Access to Care for the Homeless
EYS: Emergency youth shelter
ICHA: Inner City Health Associates
YEH: Youth experiencing homelessness
